# Comparison of the Antibacterial Efficacy of Bamboo Shoot Ethanol Extract With Chlorhexidine Mouth Rinse Against Salivary Streptococcus mutans and Lactobacillus acidophilus: An Ex Vivo Study

**DOI:** 10.7759/cureus.53085

**Published:** 2024-01-28

**Authors:** Divya Bharathi S, Priya Deepa Lakshmi K, Gunasekaran M, Venkata Lakshmi S, Anjali Reji, Kathija Sulthana F

**Affiliations:** 1 Dentistry, Vinayaka Mission’s Sankarachariyar Dental College, Vinayaka Mission’s Research Foundation (Deemed to be University), Salem, IND; 2 Public Health Dentistry, Vinayaka Mission's Sankarachariyar Dental College, Vinayaka Mission’s Research Foundation (Deemed to be University), Salem, IND; 3 Orthodontics and Dentofacial Orthopedics, Vinayaka Mission’s Sankarachariyar Dental College, Vinayaka Mission’s Research Foundation (Deemed to be University), Salem, IND; 4 Public Health Dentistry, Vydehi Institute of Dental Sciences and Research Centre, Bangalore, IND; 5 Public Health Dentistry, Vinayaka Mission’s Sankarachariyar Dental College, Vinayaka Mission’s Research Foundation (Deemed to be University), Salem, IND

**Keywords:** streptococcus mutans, lactobacillus acidophilus, chlorhexidine mouthrinse, bamboo shoots, antibacterial

## Abstract

Background

Dental caries is the most prevalent polymicrobial oral infectious disease tormenting individuals' healthy lifestyles and presents a significant public health problem. The objective of this study was to evaluate and compare the antibacterial properties of different concentrations of bamboo shoot ethanol extract with chlorhexidine mouth rinse on isolates of salivary *Streptococcus mutans (S. mutans*) and *Lactobacillus acidophilus *(*L. acidophilus*)*.*

Materials and methods

Non-stimulated salivary samples from 30 young adults were treated ex vivo with bamboo shoot ethanolic extract at concentrations of 30 µg/ml, 40 µg/ml, 50 µg/ml, and 60 µg/ml. The colony-forming units were quantified by measuring the number of viable bacterial cells. Inhibition zones were evaluated using the agar diffusion method. One-way ANOVA and post-hoc test were used to analyze the significant difference between variables using SPSS version 22.0 (IBM Corp., Armonk, NY).

Results

The mean zone of inhibition with bamboo shoot ethanolic extract against salivary *S. mutans* (23.00 ± 0.816) and *L. acidophilus* (22.00 ± 0.816) total counts was closest to the control chlorhexidine (*S. mutans = *22.00 ± 0.876 and *L. acidophilus = *21.10 ± 0.876). A greater activity against *S. mutans* and *L. acidophilus* is seen in the zone of inhibition of the 60 µg/ml experimental concentration of bamboo shoot ethanolic extract, with a significant difference in the disc diffusion assay.

Conclusion

The treatment with bamboo shoot extract was equivalent effective in the mentioned bacterial species.

Clinical relevance

It can be assured that preventive measures like mouth rinse and dentifrices compromising bamboo shoots, a potential dental biomaterial, would be optimistic agents for caries control, including the cariostatic effect.

## Introduction

Dental caries is the most prevalent polymicrobial oral infectious disease tormenting individuals' healthy lifestyles and presents a significant public health problem [1,2]. Globally, untreated caries affects 573 million children and 2.5 billion adults, which has a consequential negative impact on society and the healthcare system [3]. Therefore, to downsize the burden, one must first comprehend the etiology of caries, with a primary emphasis on oral microbiology and its dysbiosis followed by the practice of good preventive strategies.

Over the last several years, numerous antibiotics using natural products have been discovered due to their proven scientifically evident pharmacological effect and their utilization to treat various oral microbiological diseases. Bamboo (*Bambusa arundinacea*), a group of giant arborescent tree grasses, has a vital role in folk medicine due to its promising multidirectional biological properties [4]. Consequently, being a potential source of bioactive substances, bamboo renders natural immunity against varieties of bacteria [5]. Every part of the bamboo, i.e., leaves, shoots, culm sheath, and culms, is used either as nutritive food or medicine, due to its phenolic acid and flavonoid chemical constituents [4].

Contemporary studies [6,7] have shown that bamboo extracts can effectively reduce free radicals, i.e., the presence of a variety of biologically active compounds, and have anti-oxidant, anti-cancer, anti-ulcer, anti-diabetic, and anti-inflammatory properties [8]. Among the various parts, bamboo shoots have gained remarkable consideration.

Preliminary research demonstrated the presence of antibiotic activities by two novel chitin-binding proteins (Pp-AMP1 and Pp-AMP2) isolated from bamboo shoots [9]. Having a tremendous antimicrobial potential with diverse flavones and glucosides makes the bamboo shoot a rising natural medicine [10].

Based on a stringent review of the literature available to date, it was observed that there are few in-vitro studies [11,12] that were carried out to prove the antimicrobial activity of bamboo shoots and no clinical or experimental studies have assessed the antibacterial effect against oral pathogenic microbes. With this background, this study aimed to evaluate and compare the antimicrobial efficacy of bamboo shoot ethanol extract of different concentrations with chlorhexidine (CHX) mouth rinse against salivary *Streptococcus mutans* (*S. mutans*) and *Lactobacillus acidophilus* (*L. acidophilus*) pathogens.

## Materials and methods

Collection of plant materials

Juvenile bamboo shoots were procured from the Agroforestry Research Division, Salem, Tamil Nadu, India, and were presented for identification by the botanical experts. The collected bamboo shoots were rinsed in tap water and the milky white portions were sliced into pieces after the removal of the hard-rudimentary portions. Later, for 30 minutes, these fragments were boiled to eliminate the noxious content of prussic acid present in the shoot nomenclature. After draining the water, the sample was washed using distilled water and air-dried for two days at 45℃. The dried samples were ground to a fine powder using a pulverizer machine and this refined powder was stored for further analysis.

Preparation of the bamboo shoot extract

For the extraction of secondary metabolites, the bamboo shoot extract was prepared as follows: refined powder was macerated with 70% ethanol in 1:10 dilution with continuous stirring for 24 hours under dark conditions. Similarly, the powder was subjected to the second and third extractions using an ethanol mixture, and the clear filtrate was then created by filtering it through Whatman filter paper number 1 and this procedure was carried out using a Soxhlet apparatus. The filtrates were evaporated and stored at 4℃ in an airtight container. The bamboo shoot extract used in this study was selected based on a previous study [8,11]. For control, 0.2% chlorhexidine gluconate (Hexidine, ICPA Health Products Limited, Mumbai, India) mouth rinse was used.

Study design and study population

By employing a simple random sampling methodology, this ex vivo (observational) study was performed on a sample of 30 young adults (18 to 21 years) attending the outpatient department of Vinayaka Mission’s Sankarachariyar Dental College (VMSDC), Salem from January 2023 to April 2023. Participants with at least 24 natural teeth, having a Decayed, Missing, and Filled Teeth (DMFT) score of ≥ 3, and free of periodontal diseases, having a probing depth of ≤ 3 mm on clinical examination were included in the study. Potential individuals were excluded if they have any pre-existing systemic diseases and are under medication, have a history of smoking or any other personal habits, are under any antibiotic, anti-inflammatory, or corticosteroid therapy in the last two months, individuals undergoing orthodontic treatment, and with removable and fixed prosthesis. Participants with a history of use of any mouth rinse in the last one month and oral prophylaxis in the last three months were also excluded from this study.

Ethical approval

The study design was examined and approved by the Institutional Ethical Panel Committee of Vinayaka Mission’s Sankarachariyar Dental College (VMSDC/IEC/Approval No. 303).

Saliva sampling

Before the saliva sample collection, written informed consent was obtained to take part in the study. Participants were instructed not to consume/eat food or beverages, except for water one hour before saliva collection. Therefore, the non-stimulated saliva samples from the study population were collected by spitting into a graduated sterile tube. Saliva produced in the first 30 seconds was discarded and then it was collected for exactly five minutes (quantity: 3 ml). Then the collected samples were stored in the insulated ice box (4°C) and within one hour transferred to the microbiology laboratory for further analysis.

Microbial testing

The collected samples were spread on nutrient agar Petri dishes after diluting saliva (1:10) with 1 ml of sterile peptone water. Later, these dishes were incubated at 37℃ for 48 hours. Active colonies were prepared by transferring a loopful of cells from the stock cultures to the test tube of Mueller Hinton broth. The Mueller Hinton agar plates were prepared by pouring 15 ml of molten media into sterile Petri dishes. The plates were allowed to solidify for five minutes and 0.1% inoculum suspension was swabbed uniformly and this was allowed to dry for five minutes. The disc diffusion method was used to screen the antimicrobial activity. The different concentrations (30 µg/ml, 40 µg/ml, 50 µg/ml, and 60µg/ml) of extracts and control mouth rinse were loaded (40 µg/disc) on a 6 mm sterile disc. The loaded discs were placed on the surface of the medium and the extract was allowed to diffuse for five minutes and the plates were kept for incubation at 37℃ for 24 hours. At the end of incubation, inhibition zones formed around the disc were measured using a transparent ruler in millimeters.

Statistical analysis

The results were analyzed using the IBM SPSS Statistics for Windows, version 22.0 (IBM Corp., Armonk, NY). One-way ANOVA was used to analyze the significant difference, and a follow-up test called a post-hoc test was performed to assess the significant difference between the variables. For all tests, a difference was deemed statistically significant if the p-value was ≤ 0.05.

## Results

**Figure 1:**
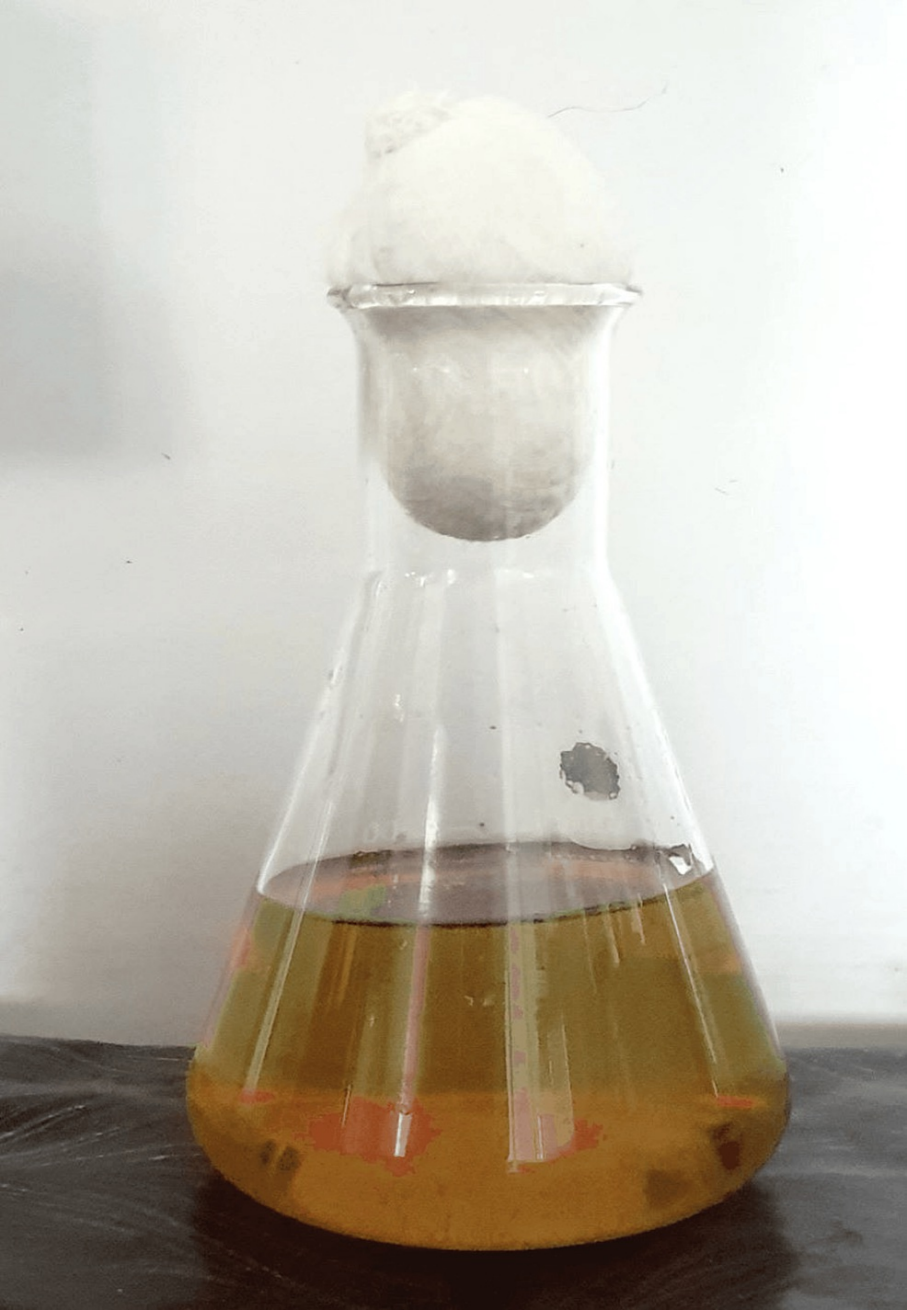
Bamboo shoot ethanolic extract solution.

In the current study, organoleptic testing revealed that the extract solution had a strong earthy smell and a clear golden-brown appearance (Figure 1).

**Figure 2:**
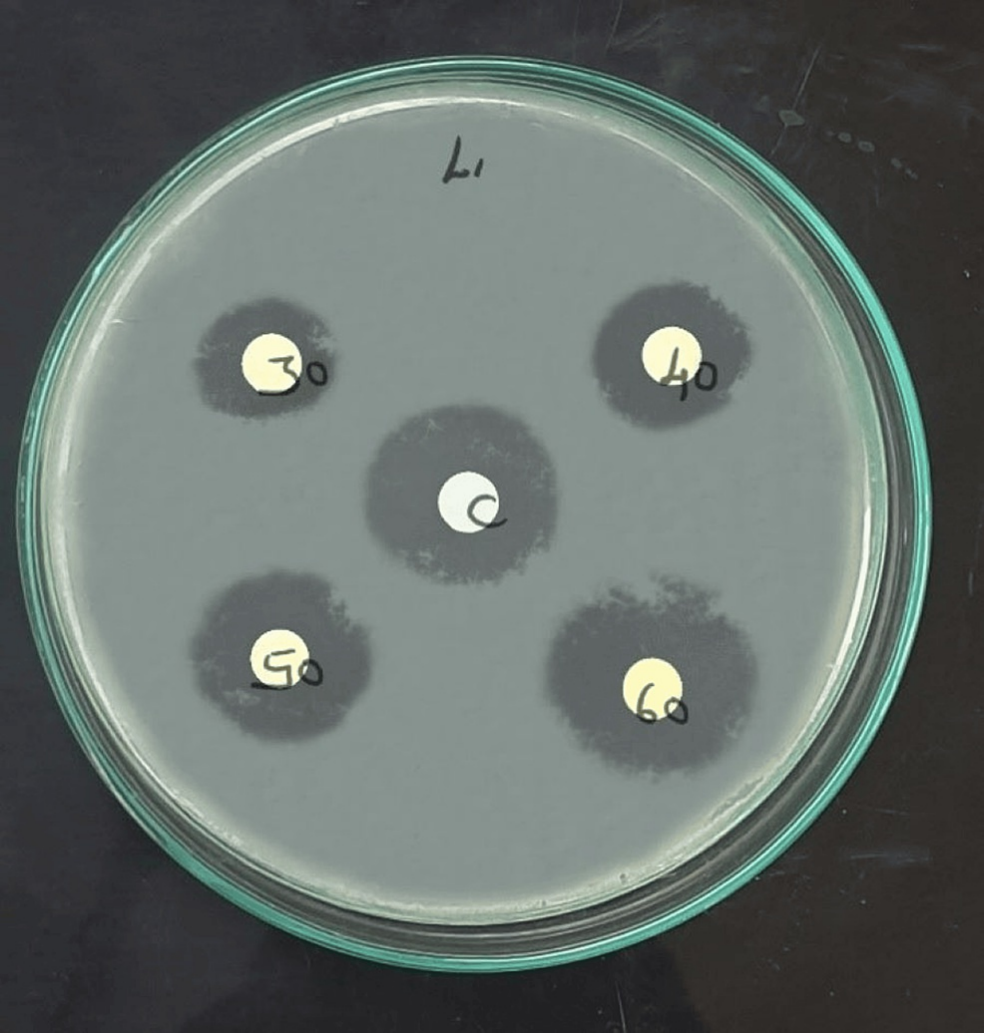
Bamboo shoot ethanolic extract and chlorhexidine mouth rinse inhibition zone of Streptococcus mutans at varied concentrations (30, 40, 50, and 60 µg/ml) in Mueller Hinton agar.

**Figure 3:**
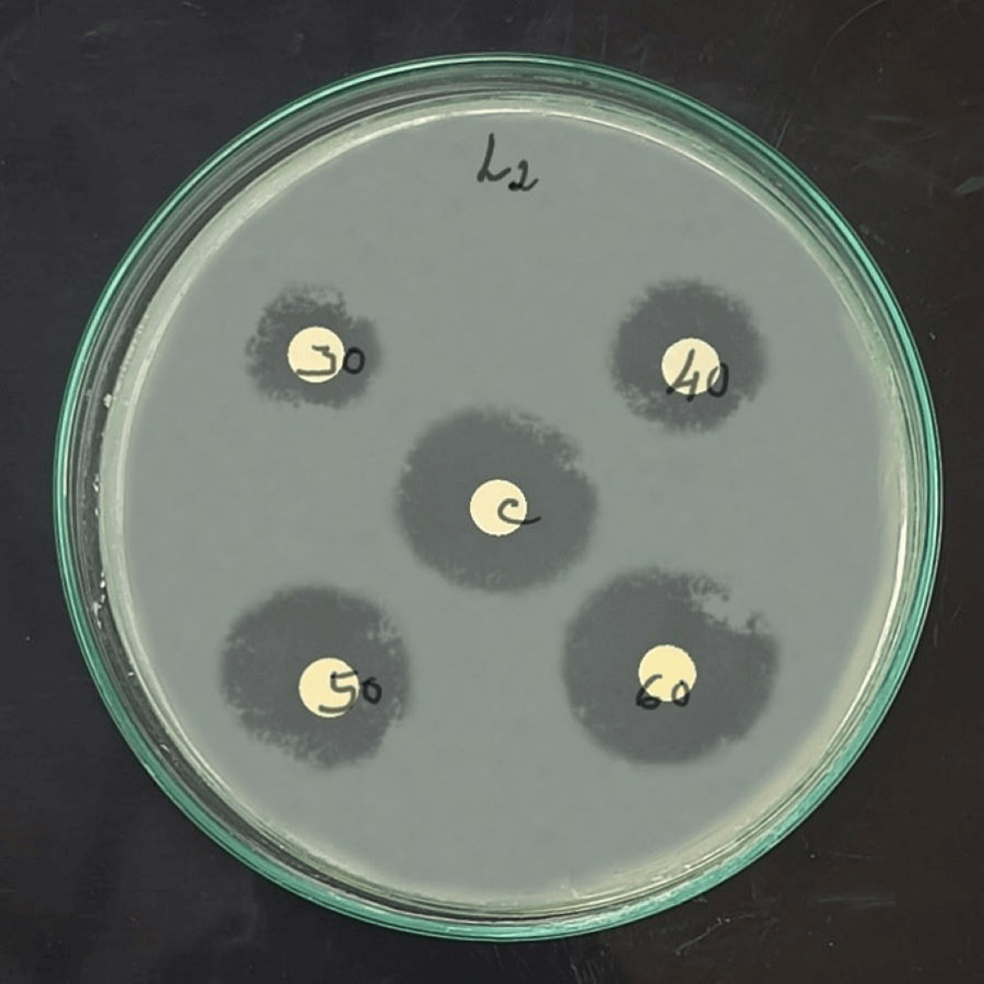
Bamboo shoot ethanolic extract and chlorhexidine mouth rinse inhibition zone of Lactobacillus acidophilus at varied concentrations (30, 40, 50, and 60 µg/ml) in Mueller Hinton agar.

Figures 2, 3 showed that the antimicrobial activity of bamboo shoot ethanol extract with the control CHX against *S. mutans* and *L. acidophilus* was quantitatively assessed by inhibition zones.

The mean zone of inhibition of bamboo shoot ethanolic extract and 0.2% chlorhexidine against salivary *S. mutans* and *L. acidophilus* is shown in Table 1.

**Table 1:** Mean zone of inhibition of bamboo shoot ethanolic extract and 0.2% chlorhexidine mouth rinse against salivary Streptococcus mutans and Lactobacillus acidophilus.

Variable	Streptococcus mutans (mean ± SD)	Lactobacillus acidophilus (mean ± SD)
30 µg/ml concentration extract	13.20 ± 1.317	13.50 ± 1.354
40 µg/ml concentration extract	18.00 ± 0.816	17.00 ± 0.816
50 µg/ml concentration extract	20.00 ± 0.816	19.90 ± 0.876
60 µg/ml concentration extract	23.00 ± 0.816	22.00 ± 0.816
0.2% chlorhexidine	22.00 ± 0.876	21.10 ± 0.876

A comparison of the shoot extract and mouthwash with the mean inhibitory zone and the susceptibility pattern of the control with bamboo shoots against *S. mutans* is displayed in Table 2.

**Table 2:** Descriptive statistics of the disc diffusion assay results of bamboo shoot ethanol extract with 0.2% chlorhexidine (control) against Streptococcus mutans. * p-value ≤ 0.05.

Variable		Mean difference	Standard error	P-value	95% confidence interval	
					Lower bound	Upper bound
0.2% chlorhexidine (control)	30 µg/ml concentration extract	-1.500	0.416	0.005*	12.26	14.14
	40 µg/ml concentration extract	0.500	0.258	0.308	15.42	16.58
	50 µg/ml concentration extract	0.250	0.258	0.115	19.42	20.58
	60 µg/ml concentration extract	0.000	0.258	0.047*	22.42	23.58

A greater activity against *S. mutans* is seen in the zone of inhibition of the 60 µg/ml experimental concentration of bamboo shoot ethanolic extract, which also demonstrated a significant (p = 0.047) difference in the disc diffusion assay. With a mean difference of -1.500, the 30 µg/ml concentration of extract demonstrated a notable significant variance, indicating that the zone of inhibition is less than that of the control.

According to Table 3 findings, there was a statistically significant (p = 0.036) difference between 60 µg/ml concentrations against *L. acidophilus*.

**Table 3:** Descriptive statistics of the disc diffusion assay results of bamboo shoot ethanol extract with 0.2% chlorhexidine (control) against Lactobacillus acidophilus. * p-value ≤ 0.05.

Variable		Mean difference	Standard error	P-value	95% confidence interval	
					Lower bound	Upper bound
0.2% chlorhexidine (control)	30 µg/ml concentration extract	-1.000	0.428	0.694	12.53	14.47
	40 µg/ml concentration extract	0.000	0.258	0.906	16.42	17.58
	50 µg/ml concentration extract	0.000	0.277	0.144	19.27	20.53
	60 µg/ml concentration extract	-1.167	0.258	0.036*	22.42	23.58

## Discussion

This research has proclaimed the potential application of bamboo shoots in controlling dental caries since it has already been incorporated into various commercial and domestic purposes. A sequence of scientific work has been performed exploring the antimicrobial properties of shoot extract on different microorganisms [13,14]. To the best of our cognizance from the existing data, the activity of these extracts on cariogenic commensals is deficient. As a result, our analysis is the primary to address the antimicrobial activity of shoot extract against oral cavity-derived bacteria.

The authors believe that in the present qualitative assay conditions, the phytochemical constituents such as alkaloids, phenols, flavonoids, and saponins provoked the inhibitory activity against the salivary microbes and this was in accordance with the previously published literature on bamboo shoots by Ren et al. [15]. The null hypothesis was rejected, as there was a significant difference in the antimicrobial effects between the 60% concentration of the tested and control group against *S. mutans*.

A succession of studies [16-18] have looked into the antibacterial action of CHX, and our results are in line with the scientific evidence that has been published. An in vitro study that discovered substantial inhibitory zones linked to different concentrations of bamboo extract further supported the anti-adhesion action [19]. The post-hoc tests revealed a significant difference between CHX and the particular concentrations of bamboo shoot extract against *S. mutans* and *Lactobacillus* organisms.

Evidence indicating the anticancer, antioxidative, antibacterial, and anti-inflammatory properties of bamboo is strongly supported by the literature [20-22]. Behera and Balaji [23] did not discover any microbial growth in fermented bamboo shoots during an investigation into the processing and preservation.

One of the variables examined in the present study was the effect of the mouthwash and shoot ethanol extract on the diameter of the zone of inhibition. Earlier in vitro studies by Ramful et al. [5] have affirmed that bamboo plant extract in ethanol provided more consistent antimicrobial potential. Moreover, in a study conducted by Sharma et al. [24], *Calotropis gigantea*, a natural habitat of tribal people of Asian countries, disrupted the growth of *S. mutans* and lactobacilli with a clear zone of 16 mm and 14 mm around the disc.

The present study has some limitations that should be considered in future investigations of this topic. More relevant clinical studies and in vivo studies are needed to establish quality control protocols for bamboo shoot-containing agents. In this study, the investigations were performed on a small cohort of participants. With the aid of a similar experimental setup and considering several intraoral factors, a larger sample could be obtained for further investigations with bamboo shoot ethanolic extract. Also, a determination of whether there is efficacy against other oral species, such as periodontal pathogens, and whether this *Bambusa* extract can be used as a natural substance in the prevention of periodontitis, is needed.

From this study, we recommend using the 60% concentration of bamboo shoot ethanolic extract rinse with an approximate quantity of 10 ml in 1:1 dilution for 30 seconds to swiss intraorally after brushing twice daily (morning and night) for two weeks. Although this bamboo shoot mouth rinse had an earthy sweet taste, quality tests can further evaluate other factors such as acceptability and its effects on oral tissue, and then be carried out in vivo design as no experimental study was carried out on study participants.

## Conclusions

In the discipline of dentistry, discovering ingredients with antimicrobial qualities is a challenge. In the present study, both CHX and the bamboo shoot extract were equivalent effective in the *S. mutans* and *L. acidophilus* bacterial species in ex vivo testing. It can be concluded that preventive measures like mouth rinse and dentifrices comprising bamboo shoots, a potential dental biomaterial, would be optimistic agents for caries control, including the cariostatic effect. Prospects of the current investigations should be done on bamboo shoots' biological properties to determine how the ethanol extract of bamboo shoots interacts with other adjuvants and active anti-cariogenic compounds. To corroborate clinical findings in terms of different bacterial habitats, a more in vivo experimental study considering other intraoral factors is also required.
